# Dietary Strawberries Improve Serum Antioxidant Profiles in Adults with Prediabetes: A 28-Week Randomized Controlled Crossover Trial

**DOI:** 10.3390/antiox14101258

**Published:** 2025-10-20

**Authors:** Shauna Groven, Pamela Devillez, Robert Hal Scofield, Amber Champion, Kenneth Izuora, Arpita Basu

**Affiliations:** 1 Department of Kinesiology and Nutrition Sciences, University of Nevada, Las Vegas, NV 89154, USA; groven@unlv.nevada.edu (S.G.); pdeville@unlv.nevada.edu (P.D.); 2 Division of Endocrinology, Department of Internal Medicine, Kirk Kerkorian School of Medicine, University of Nevada, Las Vegas, NV 89102, USA; amber.champion@unlv.edu (A.C.); kenneth.izuora@unlv.edu (K.I.); 3 Arthritis & Clinical Immunology, Oklahoma Medical Research Foundation, Oklahoma City, OK 73104, USA; hal-scofield@omrf.org

**Keywords:** strawberry, prediabetes, antioxidants, glutathione, oxidative stress, glycemic control

## Abstract

Prediabetes increases oxidative stress and the risk of type 2 diabetes and related cardiovascular diseases. Previous trials have shown antioxidant-rich strawberries improve this risk, but effects on antioxidant markers are inconclusive. This 28-week randomized controlled crossover trial evaluated the effects of freeze-dried strawberries (FDS) on fasting glucose, serum antioxidant status, and vascular inflammation in adults with prediabetes not on glucose-lowering medications. Participants were assigned to FDS (32 g/day ~ 2.5 servings of whole strawberries) or control (usual diet, no strawberries) for 12 weeks each, separated by a 4-week washout (n = 25/treatment period). Biomarkers were measured at baseline, 12, 16 (baseline 2), and 28 weeks. A mixed-model analysis of variance detected differences between groups, adjusting for covariates. Compared to control, FDS significantly improved serum superoxide dismutase (0.08 ± 0.04 U/mL), glutathione [(GSH): 1.8 ± 0.96 µmol/L], antioxidant capacity [(AC): 5.9 ± 3.2 µmol/L], β-carotene (113.9 ± 15.8 nmol/L), fasting glucose (97 ± 12 mg/dL), intercellular adhesion molecule [(ICAM): 56.0 ± 21.8 ng/mL], and vascular cell adhesion molecule [(VCAM): 440 ± 163 ng/mL] (all *p* < 0.05). ICAM was inversely correlated with GSH (r = −0.21), AC (r = −0.15), and β-carotene (r = −0.13) (all *p* < 0.05). VCAM was inversely correlated with AC (r = −0.12) (*p* < 0.05). Catalase, glutathione reductase, glutathione peroxidase, α-carotene, P-selectin, and E-selectin were unaffected. Our findings support strawberry intake as a dietary intervention for improving blood glucose control and antioxidant status in adults with prediabetes.

## 1. Introduction

Prediabetes, characterized by elevated fasting plasma glucose (FPG) below the threshold for type 2 diabetes mellitus (T2DM), is a critical stage where dietary interventions can prevent further hyperglycemia and disease progression [1]. Hyperglycemia increases oxidative stress due to an imbalance of reactive oxygen species (ROS) and antioxidant capacity (AC), thereby impairing insulin secretion and driving insulin resistance (IR) [2]. Chronic inflammation also promotes IR by increasing tumor necrosis factor-α (TNF-α), a proinflammatory cytokine elevated in T2DM, which inhibits glucose transport by reducing glucose transporter type 4 (GLUT4) [3,4]. In prediabetes and TD2M, increased oxidative stress damages cells from excessive free radicals, which elevate inflammation, creating a cycle of ROS generation and inflammatory responses. TNF-α also activates inflammatory pathways (c-Jun N-terminal kinase and nuclear factor-kappa B), reducing insulin-stimulated glucose uptake and altering ROS–antioxidant balance [5,6]. Therefore, upregulation of proinflammatory markers, like interleukin-13 and C-reactive protein (CRP), has been linked to disease progression [7,8].

Hyperglycemia also promotes endothelial dysfunction through several mechanisms: increased free radical production (superoxide and hydrogen peroxide); downregulation of antioxidant enzymes, as observed in human umbilical vein endothelial cells (HUVECs) [9]; and upregulation of cellular adhesion molecules (CAMs), such as ICAM and VCAM. These changes recruit leukocytes during inflammation and initiate atherosclerosis, the most common macrovascular T2DM complication [10,11,12]. In addition, injured endothelial microparticles in diabetes increase NADPH oxidase, promoting vascular inflammation via ROS [13]. Antioxidant enzymes, such as superoxide dismutase (SOD) and catalase (CAT), are critical in preventing endothelial cell damage by neutralizing ROS.

Dietary antioxidants, such as vitamins C and E, carotenoids, and polyphenols, have been identified as potential interventions to reduce oxidative stress and T2DM risk. While some studies question the relationship between dietary antioxidants and antioxidant biomarkers due to reduced absorption or phytochemical modification [14], a meta-analysis found plant-derived treatments significantly improved oxidative stress, total antioxidant capacity (TAC), and antioxidant enzyme activity compared to placebos or pharmacological treatments in T2DM [15]. Xu et al. showed a higher oxidative balance score (OBS), derived from dietary antioxidant exposure and lifestyle factors, negatively correlated with all-cause and cardiovascular mortality in adults with prediabetes, indicating a meaningful benefit in this high-risk group [16]. Concerning antioxidant status, one study found that adults with prediabetes had decreased nuclear factors (erythroid-derived 2)-like 2 (Nrf2), a transcription factor that activates antioxidant enzymes [17]. Sireesh et al. reported that pterostilbene, an antioxidant in berries and nuts, activated Nrf2, leading to improved glucose homeostasis, decreased proinflammatory cytokines, and inhibition of pancreatic β-cell apoptosis in diabetic mice [18]. Randomized controlled trials (RCTs) also show dietary antioxidants, such as Eriomin^®^ (a lemon flavonoid extract), astaxanthin (a carotenoid in algae), saffron (a carotenoid-rich spice), and curcumin (a turmeric polyphenol), improve glycemic control and oxidative stress markers in prediabetes, including insulin sensitivity, IR, FPG, 2 h postprandial glucose, HbA1c, CRP, TNF-α, and AC [19,20,21,22].

Berry fruits exhibit potent antioxidant properties as they are rich in polyphenols, such as anthocyanins and ellagic acid. Many studies show berry intake improves oxidative stress and TAC in metabolic syndrome (MetS), which includes hyperglycemia, though results vary by berry type and study duration [23]. Another systematic review reported 32% of 56 oxidative stress biomarkers improved after berry consumption [24]. Nair et al. demonstrated blueberry intake reduced ROS and proinflammatory cytokines in MetS [25], while another RCT found strawberry and cranberry beverages did not significantly change oxidative stress, but improved insulin sensitivity in overweight or obese non-diabetic, insulin-resistant adults [26]. Our previous studies showed 14 weeks of 32 g/day freeze-dried strawberry (FDS) supplementation reduced fasting insulin and homeostatic model of assessment of IR (HOMA-IR) [27], increased AC and SOD, and decreased VCAM-1 and TNF-α [28]. In our study of obese adults with hyperlipidemia, 12 weeks of 25 g/day FDS improved AC, glutathione (GSH), and CAT [29]. While these findings support improved antioxidant defenses and insulin responses, further research should assess whether FDS yields similar benefits if glucose metabolism is altered. To address this knowledge gap, our current study examined whether FDS supplementation affects serum antioxidants and vascular inflammation in individuals with prediabetes. We measured various antioxidant markers to evaluate therapeutic potential: CAT and SOD (ROS neutralizers); α- and β-carotene (dietary antioxidants); AC (total antioxidant effect); and GSH, glutathione reductase (GR), and glutathione peroxidase (GPX), which maintain redox balance. CAMs (ICAM, VCAM, P-selectin, and E-selectin) were also measured to assess inflammation. Given evidence suggesting antioxidant-rich foods can reduce oxidative stress and metabolic dysfunction, we hypothesized that FDS consumption would significantly increase antioxidant biomarkers and reduce vascular inflammation compared to control over 12 weeks. Considering oxidative stress drives prediabetes progression, this study explores a non-pharmacological dietary approach to mitigate free radical damage and improve glycemic control [30].

## 2. Materials and Methods

### 2.1. Participants

This is a randomized controlled crossover trial conducted at the diabetes clinic at the UNLV School of Medicine between July 2022 and June 2024. We enrolled a total of 25 participants, with each completing both the intervention and control periods in a randomized crossover design. Inclusion criteria comprised adults meeting the American Diabetes Association criteria for prediabetes (fasting plasma glucose 100–125 mg/dL, HbA1c 5.7–6.4%, or 2 h plasma glucose 140–199 mg/dL following a 75 g oral glucose tolerance test). Additional criteria required that men have a waist circumference greater than 40 inches and women have a waist circumference greater than 35 inches, and insulin resistance determined by HOMA-IR ≥ 2.0 as a risk factor for T2DM [31,32]. Exclusion criteria included participants who were on glucose-lowering medications, weight-loss diets, pregnant or lactating, taking herbal supplements, allergic to strawberries, or were not able to provide consent to participate in the study. Written informed consent was obtained from all participants, and the study was registered at clinicaltrials.gov (NCT05362968). The study was approved by the UNLV ethics committee (Study number, year: 1337947, 2022).

### 2.2. Intervention and Study Design

This was a 28-week randomized controlled crossover trial in which each participant was randomized to the FDS intervention or ‘no FDS’ control group, each for 12 weeks, followed by a four-week washout phase, and then they switched to the second phase for 12 weeks until completion. Randomization was performed using an SAS-generated randomization version 9.4 order of two intervention codes, and the research coordinator implemented the process. The intervention group received 32 g FDS powder (~2.5 servings of fresh strawberries) to be consumed in two daily doses of 16 g each, reconstituted in plain drinking water. Based on our prior work using a similar powder representing ~10% fresh weight, ~360 g of fresh strawberries (2.5 servings) corresponds to 32 g freeze-dried powder, given that strawberries are ~91% water by weight [33,34]. The powder was provided by the California Strawberry Commission (Watsonville, CA, USA). The daily dose provided the following nutrients and bioactive compounds: 125 kcal, 784 mg total polyphenols, 72 mg total anthocyanins, 166 mg total flavonols, and 5.5 g dietary fiber. Both groups followed their usual diet and lifestyle habits during the 12-week period, with the intervention group receiving FDS supplementation and the control group receiving no dietary interventions. Height and body weight were determined, and blood samples were drawn at baseline, 12 weeks, 16 weeks (end of washout, new baseline), and 28 weeks of the study for each participant in the fasting state. The 4-week washout duration is justified by previous crossover studies in dietary strawberries that implemented a washout duration in the range of 2 to 4 weeks [35,36,37]. We selected the longer duration for maximal washout, which was confirmed by the detection (or absence) of plasma ellagic acid as an indicator of FDS intake at the end of the washout period (baseline 2). Compliance was assessed based on dietary data on the timing of the FDS drink consumed, return of unused powder, and plasma ellagic acid concentrations as an objective marker of FDS adherence. Habitual dietary fruit and vegetable consumption was determined using the ESHA’s Food Processor nutrition analysis software version 11.4 for each participant. We also collected information on baseline physical activity, medications, and multivitamin usage from participants in the study.

### 2.3. Biochemical Analyses

Freshly drawn blood samples were sent to Quest Diagnostics (Las Vegas, NV, USA) for analysis of fasting serum glucose. Serum adhesion molecules (ICAM, VCAM, E-selectin, and P-selectin) were determined using ELISA kits (R&D Systems, Minneapolis, MN, USA) based on the manufacturer’s protocol (Quantikine, R&D Systems). Serum alpha- and beta-carotene were measured using the high-performance liquid chromatography (HPLC) method as previously described by Karppi et al. [38]. Serum catalase was measured using Catalase-520 (Oxis Research, Portland, OR, USA) spectrophotometric assay based on the manufacturer’s protocol. GPX was measured by using GPx-340 (Oxis Research) based on the manufacturer’s protocol. GR and SOD activity were measured using commercially available kits (Cayman Chemical, Ann Arbor, MI, USA) in accordance with the manufacturer’s protocol. Reduced GSH was measured, as previously described by Beutler et al. [39], based on the absorbance of the yellow thiolate anion at 412 nm. Serum AC was measured using the metmyoglobin assay developed by Miller et al. [40]. The AC is based on the scavenging of the 2,2′-azinobis-(3-ethylbenzothiazoline-6-sulfonic acid) radical, converting it into a colorless product in presence of the antioxidant strawberry compounds [41]. All assays were performed in triplicate, and the average inter-assay coefficients of variation ranged between 4.2% and 6.8%.

### 2.4. Statistical Analyses

Data were screened for outliers, and descriptive statistics for continuous outcomes were summarized as means ± SD. To examine the effects of ‘FDS’ vs. ‘no FDS’ treatment on antioxidant and vascular inflammatory variables, we used a mixed-model analysis of variance (ANOVA), adjusting for the fixed effects of treatment, period, randomization order, age, sex, fasting blood glucose over time, and each variable’s baseline value. Residual normality for each model was evaluated by examining skewness, with values between –2 and +2 considered acceptable based on established criteria [42]. All models exhibited skewness within this range, indicating assumptions for mixed-model ANOVA were met. Treatment-by-period interactions were tested for possible carryover effects between intervention phases, with significant period effects retained in the final model. We also examined partial correlation coefficients for any significant correlations among the serum antioxidant and vascular inflammatory biomarkers, adjusting for age, fasting glucose, and each variable’s baseline value in the pooled sample. We had >80% power to detect differences in serum SOD (effect size 0.98), GSH (effect size 1.03), and AC (effect size 0.75) using a sample size of at least 16 adults per treatment in a two-tailed paired-samples design, as determined from our prior dose–response study of FDS [28]. Similarly, in our previous trial on the effects of FDS on adhesion molecules [28], we had >80% power to identify changes in serum VCAM (effect size 0.87) using a sample size of at least 20 adults per treatment phase in a two-tailed paired-samples design. Multiple-hypotheses testing was corrected using the false discovery rate, and statistical significance was defined as *p* < 0.05. All analyses were conducted with SAS version 9.4 (SAS Institute Inc., Cary, NC, USA).

## 3. Results

### 3.1. Baseline Features and Compliance

As summarized in Table 1, participants who completed the study were predominantly female, self-reported Hispanic, and classified as obese according to BMI. No differences in baseline characteristics were observed between randomization sequences. Study compliance exceeded 85% among participants who completed the study based on return of unused FDS powder, review of dietary logs, and plasma ellagic acid. Plasma ellagic acid was absent at baseline, washout, and 6- and 12-week control phases, but became measurable at 6 and 12 weeks of FDS consumption (27.6 ± 9.1 ng/mL and 30.2 ± 8.8 ng/mL, respectively).

### 3.2. Serum Antioxidant Markers and Fasting Glucose

The FDS group showed significant improvements in several antioxidant markers and fasting glucose relative to the control (no FDS) group (Table 2). After adjusting for treatment, period, randomization order, age, sex, fasting blood glucose over time, and each variable’s baseline value, the absolute adjusted means of serum SOD, GSH, AC, and β-carotene were 0.1 ± 0.04 U/mL, 1.8 ± 0.96 μmol/L, 5.9 ± 3.16 μmol/L, and 113.9 ± 15.82 nmol/L, respectively, in the FDS group (all *p* < 0.05). FDS treatment did not significantly affect serum catalase, GR, GPX, and α-carotene concentrations. Additionally, FDS significantly improved fasting glucose versus the control, with an absolute adjusted mean of 97 ± 12.24 mg/dL (Table 2). The effect size estimates (partial eta squared) from mixed-model ANOVA for treatment effect were as follows: catalase: 0.08; SOD: 0.32; GSH: 0.41; GR: 0.03; GPX: 0.12; AC: 0.48; α-carotene: 0.11; β-carotene: 0.48; glucose: 0.67.

### 3.3. Serum Markers of Vascular Inflammation

The FDS intervention significantly affected vascular inflammatory markers relative to the control (no FDS) group (Table 3). After adjusting for treatment, period, randomization order, age, sex, fasting blood glucose over time, and each variable’s baseline value, the absolute adjusted means of serum ICAM and VCAM were 56.0 ± 21.8 ng/mL and 440.6 ± 162.6 ng/mL, respectively. No significant differences were noted in serum P-selectin and E-selectin following the FDS period. The effect size estimates (partial eta squared) from the mixed-model ANOVA for treatment effect were as follows: ICAM: 0.43; VCAM: 0.56; P-selectin: 0.13; and E-selectin: 0.09.

### 3.4. Partial Correlation Coefficients Among Serum Antioxidants and Vascular Inflammatory Biomarkers

Partial correlations were examined between several serum antioxidants (SOD, GSH, AC, and β-carotene) and vascular inflammatory biomarkers after adjusting for age, fasting blood glucose, and baseline value for each outcome. Serum ICAM was inversely correlated with serum GSH (r = −0.21), AC (r = −0.15), and β-carotene (r = −0.13) (all *p* < 0.05), while its correlation with SOD was not significant. Serum VCAM was significantly inversely correlated with serum AC (r = −0.12), but no other serum antioxidants. Serum P-selectin and E-selectin had no significant correlations with serum antioxidants (Table 4).

### 3.5. Side Effects

Overall, participants experienced few side effects from FDS treatment, with two reporting gastrointestinal disturbances and one reporting headaches. These potential adverse effects were expected and disclosed to participants during the informed consent process. As all participants had prediabetes and they were clinically monitored throughout the study, with no glycemic abnormalities observed.

## 4. Discussion

Overall, our study demonstrated that 12 weeks of FDS at a dietary-achievable dose significantly improved antioxidant status, glucose control, and vascular inflammation in individuals with prediabetes—a novel finding for this population, as no previous study has utilized a randomized controlled crossover design. Previous work has shown that daily consumption of two low-calorie fruits for 3 months improved oxidative stress and glycemic status in T2DM [43]. Strawberries are particularly relevant as they are the third most valuable U.S. fruit crop, accounting for 13% of production value [44]. Furthermore, consumer data indicate 52% of U.S. households purchase fresh strawberries annually [45], and they appear on over half of restaurant menus nationwide, more than any other berry [46]. Research has also highlighted their role in supporting overall dietary quality and health, with strawberries identified among the top 10 antioxidant-rich foods consumed in the U.S. [47]. Analysis of data from 2003 to 2018 further demonstrated that approximately 25% of U.S. adults consume berries, with strawberries being the most common, and that berry intake is associated with lower cardiometabolic risk [48]. Globally, strawberry production exceeds millions of tons annually, with Asia, the Americas, and Europe as the leading producers, reinforcing their widespread presence and nutritional significance [49]. Since T2DM has been associated with increased oxidative stress, the observed significant differences in serum antioxidants and adhesion molecules in our study are also of clinical importance. While not routinely assessed in the clinical prevention and management of prediabetes and T2DM, reported clinical data show serum antioxidant enzymes, such as lower SOD activity, and lower serum carotenoids are predictive of insulin resistance and beta cell failure, thereby increasing diabetes risk [50,51,52]. Furthermore, a meta-analysis shows increased circulating levels of ICAM-1 and E-selectin are associated with increased risk of T2DM in prospective studies [53]. Collectively, our data on the role of strawberries in improving these clinically important biomarkers of antioxidant and endothelial dysfunction support strawberries as a practical dietary intervention for adults with prediabetes for reducing the risk of T2DM.

Considering antioxidant status, our study showed FDS supplementation significantly improved serum SOD, GSH, AC, and β-carotene, indicating reduced oxidative stress in individuals with prediabetes. Prior research showed that consuming 500 g/day of fresh strawberries for one month improved oxidative stress markers and total AC in healthy adults [54], but our findings extend these outcomes to those with prediabetes. Similarly, Moazen et al. reported that 50 g/day of FDS for 6 weeks improved total AC in adults with T2DM compared with a macronutrient-matched placebo, highlighting that FDS itself benefits the T2DM population, although individual antioxidant markers were not assessed [55]. Our study expands on this by demonstrating that FDS improved fasting glucose over time in prediabetes, an effect not examined in earlier trials that focused on postprandial insulin dynamics—one documented a blunted 6 h insulin response after FDS with a high-carbohydrate, moderate-fat meal in overweight adults [56], while another reported 40 g of FDS lowered 6 h insulin concentrations and insulin-to-glucose ratios after a high-carbohydrate, high-fat meal in individuals with IR [57]. These results suggest that strawberries also reduce postprandial insulin demand, even in those with dysregulated glucose metabolism. Data from our trial also indicate that FDS can significantly improve markers of vascular inflammation linked to prediabetes, as evidenced by reductions in ICAM and VCAM. In one of our prior RCTs, 50 g/day of FDS for 8 weeks in individuals with MetS lowered VCAM-1 without significantly changing ICAM-1, potentially reflecting differences in study durations and populations, as IR is not always present in MetS [58]. Another trial showed 3 weeks of FDS (equivalent to 320 g/day of frozen strawberries) had no effect on antioxidant status or CAMs in obese adults, possibly due to the lack of IR promoting increased CAMs or the shorter intervention period [37]. While prior RCTs did not investigate the effects of FDS on selectins [37,58], we found no significant changes in selectins despite reductions in CAMs. Variations across studies may also reflect differences in dose, intervention duration, and forms of strawberry supplementation.

Concerning mechanisms responsible for effects seen in our study, FDS significantly increased serum GSH without altering GR or GPX, aligning with evidence that polyphenols primarily enhance de novo GSH synthesis rather than circulating enzyme activity. Moskaug et al. showed polyphenol-mediated upregulation of γ-glutamylcysteine synthetase via Nrf2 activation [59], while Niu et al. reported increased GSH and glutathione synthetase with variable GR and GPX responses, suggesting FDS elevates GSH through synthesis rather than recycling pathways [60]. Enhancement of serum SOD by FDS indicates upregulation of endogenous antioxidant defenses [61], while elevation of AC and β-carotene reflects greater non-enzymatic and carotenoid antioxidant activity [62]. However, the absence of significant changes in catalase and α-carotene may reflect differences in enzyme distribution or absorption efficiency. Collectively, these antioxidant effects and several bioactive constituents in FDS likely contributed to the reduction in fasting glucose. Ellagic acid, quantified in our study for compliance, reduces pancreatic β-cell dysfunction and postprandial hyperglycemia [63]. Studies also show hydroxycinnamic acids, an abundant polyphenol in FDS, protect the endothelium from hyperglycemia-induced oxidative stress by lowering CAM expression [64], and anthocyanins support glucose metabolism and β-cell survival via antioxidant-mediated regulation of apoptosis [65]. While purified anthocyanins have improved HbA1c and insulin sensitivity in prediabetes [66], a meta-analysis found greater HbA1c reductions from fruit powder-derived anthocyanins, indicating synergistic actions among bioactive elements [67]. Regarding endothelial markers, an in vitro experiment showed 0.5–1 mg/mL strawberry extract decreased P-selectin and platelet aggregation, implying antithrombotic effects through suppression of inflammatory mediators [68]. In vivo, a 12-week 2.4% FDS-supplemented diet reduced vascular inflammation and opportunistic microbes in high-fat-fed mice, implicating gut microbiota mediate in the vascular benefits of FDS [69]. While selectins were unaffected by FDS in our study, likely due to their short half-life and transient role in leukocyte rolling, CAMs were significantly reduced, which is consistent with the modulation of cytokine and oxidative stress pathways by FDS polyphenols. Overall, these findings suggest FDS exerts complementary antioxidant, metabolic, and vascular effects, supporting its role in cardiometabolic health.

Our study had several strengths, including a randomized controlled crossover design to assess the causality of effects of FDS treatment on our examined biomarkers, as well as to limit interindividual variability. Furthermore, potential carryover effects were limited by a 4-week washout with new baseline measurements post-washout to reduce potential bias. We also adjusted for potential confounding factors, including treatment, period, randomization order, age, sex, fasting glucose, and each variable’s baseline value, strengthening the attribution of significant findings to FDS supplementation for observed outcomes, as these factors may have affected oxidative stress status. Additionally, the extended 12-week duration per period, longer than in most prior trials, allowed greater time for detecting significant changes. Our study participants, being adults with prediabetes and not on glucose-lowering medications, were unique, as most of the previously reported berry trials have been conducted on adults with a wide range of cardiovascular risks that did not necessarily represent impaired glucose tolerance. Importantly, a dietary-feasible FDS dose was used, unlike higher experimental doses in earlier studies, enhancing the relevance of our findings to real-world settings. We also had some limitations, including a single-blinded design with the coordinator aware of treatment allocation, and lack of participant blinding as no placebo drink was provided—this study design may have introduced expectancy or placebo effects, such as transient dietary improvements or altered adherence, although these would likely have minimal influence as our primary outcomes were objective biochemical markers measured by blinded personnel. Furthermore, the absence of a macronutrient- and fiber-matched placebo beverage introduces some uncertainty, as non-antioxidant components of the FDS beverage may have contributed to observed effects. While we collected data on diet, physical activity, and medication use, unmeasured physiological and environmental factors, such as acute illness, psychosocial stress, or environmental exposures, may have influenced our biomarkers. Self-reported dietary adherence also introduces potential social desirability and recall biases. Our study did not test multiple doses of strawberries to determine if lower doses yielded similar results, which can be addressed with dose–response experiments. Although our trial analyzed serum antioxidant markers using standardized assays widely used in nutrition research, future work should investigate red blood cell (RBC)-based antioxidant activity, which may more accurately reflect overall oxidative stress status. Intent-to-treat analysis was not possible, as some participants withdrew before the treatment period. The relatively modest sample size, while adequately powered for the primary outcomes, limited our ability to conduct subgroup analyses, including sex-stratified comparisons, due to the imbalance in enrollment (n = 6 men vs. n = 19 women). Recruitment from a single clinical site also limits generalizability, as the broader prediabetic population may have differing dietary patterns or lifestyle factors. Larger multicenter trials are warranted to confirm the reproducibility of our findings across diverse populations and evaluate potential effects between subgroups. Finally, we reduced confounding factors by excluding participants on weight-loss regimens or glucose-lowering medications; however, this limited generalizability, as many individuals with prediabetes adopt such interventions to mitigate disease progression. Future studies should, therefore, determine if FDS supplementation may be an effective dietary therapy in this population.

## 5. Conclusions

In conclusion, our findings demonstrate that a dietary-achievable dose of 2.5 servings of strawberries per day may support metabolic health in adults with prediabetes. Future research should evaluate if smaller doses provide clinical benefits, as well as if FDS supplementation enhances glucose control and slows disease progression in prediabetes and T2DM alongside pharmacological and lifestyle interventions. Considering FDS significantly improved antioxidant markers, fasting glucose, and inflammation in individuals with prediabetes, dietary strawberries can be recommended in medical nutrition therapy as a practical, nonpharmacological intervention for both prediabetes management and prevention of T2DM.

## Figures and Tables

**Table 1:** Baseline characteristics of study participants.

N	25
Age, (y)	52 ± 14
M/F (n)	6/19
Ethnicity (Hispanic %)	56
BMI, (kg/m^2^)	32 ± 2
Blood pressure medication use, n (%)	8 (32)
Antidepressant use, n (%)	6 (24)
Multivitamin use, n (%)	17 (68)
Moderate-to-vigorous physical activity, min/week *	75 (21–115)
Fruits, % recommended intake *	33 (11, 52)
Vegetables, % recommended intake *	45 (21, 63)

Data presented as means ± standard deviations (SD). * Median (interquartile range). Count data presented as n (%). Abbreviations: M = male; F = female; BMI = body mass index.

**Table 2:** Serum antioxidant markers and fasting glucose in adults with prediabetes following each treatment period in the 28-week randomized crossover study.

Variable	Baseline 1	Control (12-Week)	Baseline 2 (16-Week, Post-Washout)	FDS (12-Week)	P-Treatment (FDR-Adjusted) ^1^
Serum catalase, U/mL	40.8 ± 16.1	37.7 ± 13.9	38.4 ± 18.9	38.6 ± 15.5	0.45
Serum SOD, U/mL	0.05 ± 0.05	0.04 ± 0.03	0.05 ± 0.04	0.08 ± 0.04	**0.0002**
Serum GSH, μmol/L	1.4 ± 0.9	1.2 ± 0.7	1.2 ± 0.6	1.8 ± 0.9	**0.0003**
Serum GR, U/L	53.3 ± 21.6	51.4 ± 19.4	50.1 ± 18.1	52.5 ± 18.2	0.32
Serum AC, μmol/L	3.8 ± 3.3	3.9 ± 2.7	3.0 ± 2.2	5.9 ± 3.2	**0.01**
Serum GPX, U/L	176.4 ± 47.0	163.7 ± 50.8	171.3 ± 45.4	168.2 ± 49.8	0.17
Serum α-carotene, nmol/L	40.4 ± 16.4	44.0 ± 17.3	35.1 ± 13.9	41.2 ± 14.5	0.23
Serum β-carotene, nmol/L	106.3 ± 25.4	93.5 ± 23.3	97.9 ± 20.1	113.9 ± 15.8	**0.01**
Serum fasting glucose, mg/dL	107 ± 15	109 ± 10	116 ± 11	97 ± 12	**0.0001**

Data are presented as mean ± SD; N = 25 per treatment period. Abbreviations: FDR: false discovery rate; SOD: superoxide dismutase; GSH: glutathione; GR: glutathione reductase; AC: antioxidant capacity; GPX: glutathione peroxidase. ^1^ P for effect of treatment from MIXED procedure (SAS version 9.4; SAS Institute Inc., Cary, NC, USA), adjusted for treatment, period, order of randomization, age, sex, fasting glucose, and each variable’s baseline value. Bold indicates *p* < 0.05.

**Table 3:** Serum biomarkers of vascular inflammation in adults with prediabetes following each treatment period in the 28-week randomized crossover study.

Variable	Baseline 1	Control (12-Week)	Baseline 2 (16-Week, Post-washout)	FDS (12-Week)	P-Treatment (FDR-Adjusted) ^1^
Serum ICAM, ng/mL	72.0 ± 22.9	80.3 ± 23.3	67.6 ± 20.7	56.0 ± 21.8	**0.0002**
Serum VCAM, ng/mL	503 ± 203	528 ± 207	492 ± 198	440 ± 163	**0.01**
Serum P-selectin, ng/mL	57.4 ± 21.8	63.6 ± 23.6	53.1 ± 21.2	61.9 ± 21.8	0.21
Serum E-selectin, ng/mL	3.1 ± 2.3	3.0 ± 2.2	2.9 ± 1.8	3.0 ± 1.8	0.26

Data are presented as mean ± SD; N = 25 per treatment period. Abbreviations: FDR: false discovery rate; ICAM: intercellular adhesion molecule; VCAM: vascular cell adhesion molecule. ^1^ P for effect of treatment from MIXED procedure (SAS version 9.4; SAS Institute Inc., Cary, NC, USA), adjusted for treatment, period, order of randomization, age, sex, fasting glucose, and each variable’s baseline value. Bold indicates *p* < 0.05.

**Table 4:** Partial correlation coefficients among serum antioxidants and vascular inflammatory biomarkers adjusted for age, fasting glucose, and baseline value for each outcome in the pooled sample.

Variable	Serum SOD, U/mL	Serum GSH, μmol/L	Serum AC, μmol/L	Serum β-Carotene, nmol/L
Serum ICAM, ng/mL	−0.12 (0.25)	**−0.21 (0.004)**	**−0.15 (0.01)**	**−0.13 (0.02)**
Serum VCAM, ng/mL	−0.03 (0.11)	−0.13 (0.22)	**−0.12 (0.01)**	−0.09 (0.11)
Serum P-selectin, ng/mL	−0.01 (0.32)	0.02 (0.16)	−0.02 (0.12)	−0.04 (0.21)
Serum E-selectin, ng/mL	−0.02 (0.21)	0.01 (0.19)	−0.02 (0.17)	−0.03 (0.13)

Data presented as r (*p*-value). Abbreviations: ICAM: intercellular adhesion molecule; VCAM: vascular cell adhesion molecule; SOD: superoxide dismutase; GSH: glutathione; AC: antioxidant capacity. P for PROC CORR (SAS version 9.4; SAS Institute Inc., Cary, NC, USA), adjusted for age, fasting glucose, and each variable’s baseline value. Bold indicates *p* < 0.05.

## Data Availability

Data will be available upon request and following ethics committee approval, based on confidentiality of participant data.
